# Repeated Low-Level Blast Acutely Alters Brain Cytokines, Neurovascular Proteins, Mechanotransduction, and Neurodegenerative Markers in a Rat Model

**DOI:** 10.3389/fncel.2021.636707

**Published:** 2021-02-19

**Authors:** Lanier Heyburn, Rania Abutarboush, Samantha Goodrich, Rodrigo Urioste, Andrew Batuure, Jaimena Wheel, Donna M. Wilder, Peethambaran Arun, Stephen T. Ahlers, Joseph B. Long, Venkatasivasai Sujith Sajja

**Affiliations:** ^1^Blast-Induced Neurotrauma Branch, Center for Military Psychiatry and Neuroscience, Walter Reed Army Institute of Research, Silver Spring, MD, United States; ^2^Neurotrauma Department, Operational and Undersea Medicine Directorate, Naval Medical Research Center, Silver Spring, MD, United States

**Keywords:** blast wave-induced neurotrauma, blood-brain barrier, Piezo2 channel, TAR DNA-bindingprotein 43 (TDP-43), repetitive blast

## Abstract

Exposure to the repeated low-level blast overpressure (BOP) periodically experienced by military personnel in operational and training environments can lead to deficits in behavior and cognition. While these low-intensity blasts do not cause overt changes acutely, repeated exposures may lead to cumulative effects in the brain that include acute inflammation, vascular disruption, and other molecular changes, which may eventually contribute to neurodegenerative processes. To identify these acute changes in the brain following repeated BOP, an advanced blast simulator was used to expose rats to 8.5 or 10 psi BOP once per day for 14 days. At 24 h after the final BOP, brain tissue was collected and analyzed for inflammatory markers, astrogliosis (GFAP), tight junction proteins (claudin-5 and occludin), and neurodegeneration-related proteins (Aβ40/42, pTau, TDP-43). After repeated exposure to 8.5 psi BOP, the change in cytokine profile was relatively modest compared to the changes observed following 10 psi BOP, which included a significant reduction in several inflammatory markers. Reduction in the tight junction protein occludin was observed in both groups when compared to controls, suggesting cerebrovascular disruption. While repeated exposure to 8.5 psi BOP led to a reduction in the Alzheimer’s disease (AD)-related proteins amyloid-β (Aβ)40 and Aβ42, these changes were not observed in the 10 psi group, which had a significant reduction in phosphorylated tau. Finally, repeated 10 psi BOP exposures led to an increase in GFAP, indicating alterations in astrocytes, and an increase in the mechanosensitive ion channel receptor protein, Piezo2, which may increase brain sensitivity to injury from pressure changes from BOP exposure. Overall, cumulative effects of repeated low-level BOP may increase the vulnerability to injury of the brain by disrupting neurovascular architecture, which may lead to downstream deleterious effects on behavior and cognition.

## Introduction

Blast overpressure (BOP) exposure has become a subject of increasing concern in the Department of Defense due to increased use of breaching operations and heavy weapons systems in both combat and in training, in which military personnel are exposed to repeated low-level BOP (Tate et al., 2013). The symptomatology reported by these individuals is not typically clinically diagnosed as a concussion, due to the current lack of either sophisticated diagnostic techniques or observation by appropriately trained medical personnel (DeKosky et al., 2010; Tate et al., 2013). Transient symptoms include headache, slowed thinking, and memory deficits, and repeated exposures throughout the Warfighters’ careers may lead to chronic problems such as performing military tasks requiring critical thinking, cognitive issues, behavioral changes, and mood disorders (Tate et al., 2013; Carr et al., 2015, 2016; Kamimori et al., 2018; Sajja et al., 2019). Although blast and impact injuries are biomechanically different, some neurological symptoms of moderately-severe BOP exposure are similar to those in impact traumatic brain injury (TBI) but have been shown to have different pathophysiological profiles (Shively et al., 2016; Yamamoto et al., 2018).

Contrary to impact TBI, BOP exposure causes complex, whole-body injury, with air-filled organs such as the lungs and ears being particularly vulnerable (Sajja et al., 2020). Several studies have shown brain perturbations in the form of the disrupted blood-brain barrier (BBB) and increased brain permeability following BOP exposure (Rubovitch et al., 2012; Elder et al., 2015). In particular, tight junction proteins such as claudin-5 and occludin are altered in Warfighters’ blood-based clinical assessments following repeated BOP exposure (Duckworth, 2018). Also, glial fibrillary acidic protein (GFAP), an astrocytic marker, and vascular endothelial growth factor (VEGF), a mediator of angiogenesis in the brain, were shown to be affected following BOP exposure (Sajja et al., 2012a; Kamnaksh et al., 2014; Duckworth, 2018; Eonta et al., 2019). Similar to previous clinical and preclinical findings, we observed that exposure to BOP intensities above 13 psi for up to four exposures triggered alterations in the tight junction proteins claudin-5 and occludin, as well as alterations in neurodegeneration-related proteins and in the mechanosensitive protein Piezo2 (Heyburn et al., 2019a). The breakdown of the border separating cerebral blood flow from the brain parenchyma leaves the brain susceptible to infiltrating cells and brain-toxic elements that can promote neuroinflammation and cell death (Shetty et al., 2014).

Neuroinflammation is a major component of the injury cascade of TBI and has been the focus of many preclinical blast TBI (bTBI) studies (Ziebell and Morganti-Kossmann, 2010; Elder et al., 2015). Animal studies employing a variety of BOP exposure techniques demonstrated brain inflammation, including gliosis, neutrophil activation, and increased cytokine production [interleukin (IL)-6, interferon-γ, MCP-1, tumor necrosis factor-α (TNFα; Cernak et al., 2011; Kovesdi et al., 2011; Sajja et al., 2012b; Cho et al., 2013; Valiyaveettil et al., 2013; Kamnaksh et al., 2014; Simard et al., 2014]. Peripheral expression of pro-inflammatory cytokines, including IL-6 and TNF-α is increased in humans following moderate BOP exposure (Gill et al., 2017a, b).

A BOP wave is essentially an overpressure compression wave that can cause skull flexure, leading to increased intracranial pressure (ICP; Bolander et al., 2011). This increased ICP can potentially cause cellular and molecular perturbations, including mechanotransduction, which together may lead to subsequent deficits and symptoms (Heyburn et al., 2019a). Piezo2 is a component of mechanosensitive ion channels and acts as a pressure sensor in the brain and body, influencing neuronal function, signal transduction, and somatosensory function (Schrenk-Siemens et al., 2014; Szczot et al., 2017). Increased levels of Piezo2 in the brain could lead to increased sensitivity to pressure alterations produced by BOP exposure, lowering the threshold for deleterious signal transduction cascades and making the brain more vulnerable to low-intensity BOP (Heyburn et al., 2019a; Zhang et al., 2019).

To develop BOP injury risk criteria for brain and lungs, our group has evaluated poly-organ trauma as evidenced by lung and brain injuries and established that 8.5 psi is the threshold for lung contusion for up to 30 daily exposures and that multiple daily exposures to 10 psi BOP cause around 1% lung injury (Heyburn et al., 2019a; Sajja et al., 2020). To expand on these previous findings and to further develop head injury risk criteria associated with repeated low-level BOP exposure, several markers known to be altered in the brain from relatively high-intensity BOP exposure were evaluated in the absence of lung injury. The current study focused on exposures of 8.5 and 10 psi to define the pathophysiological response and determined how repeated primary BOP alone, with little or no injury to the lung, affects the brain. In this study, an advanced blast simulator (ABS), which closely mimics “free-field” BOP, was used to expose rats to repeated low-level BOP, with one daily exposure for 14 days. Brains were evaluated 24 h after the last blast exposure for acute alterations in markers of inflammation, BBB integrity, neurodegeneration, and pressure sensitivity to study short-term brain effects that could contribute to the pathophysiology of neurodegeneration. This information will contribute to our understanding of the consequences of repeated BOP exposure in various military training environments and the development of health hazard assessment for repeated BOP injury.

## Materials and Methods

### Animals

All animal experiments were conducted under an approved animal use protocol in an AAALACi accredited facility in compliance with the Animal Welfare Act and other federal statutes and regulations relating to animals and experiments involving animals, with strict adherence to principles stated in the Guide for the Care and Use of Laboratory Animals, NRC Publication, 2011 edition. Male Sprague–Dawley rats, 8–9 weeks old (*n* = 6 per group) that weighed ~275 g (Charles River Laboratories, Wilmington, MA, USA) were housed at 20–22°C (12 h light/dark cycle) with free access to food and water *ad libitum*. No change in weight was observed following blast exposure.

### BOP Exposure

**Figure 1:**
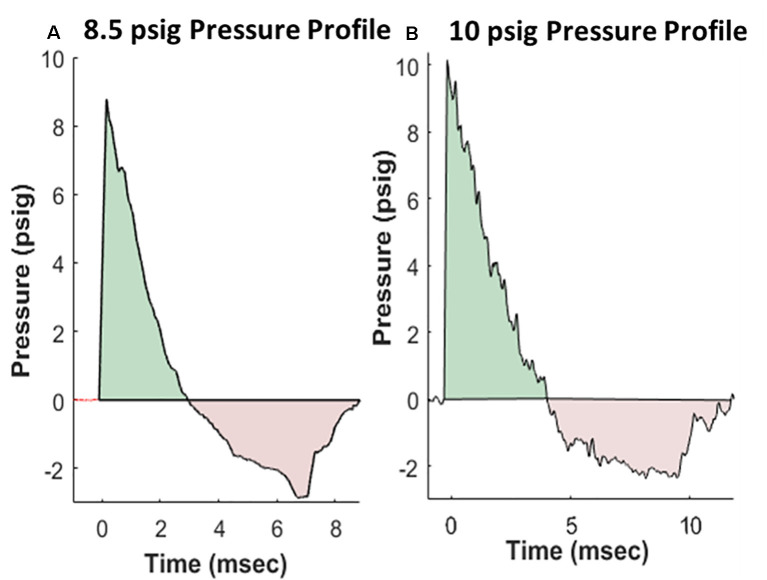
Representative pressure profiles generated using the advanced blast simulator (ABS) at Walter Reed Army Institute of Research (WRAIR) that are highly reproducible, with both positive (teal color) and negative phases (pink color) mimicking Friedlander-like “free-field” blast for the 8.5 psi **(A)** and 10 psi **(B)** groups.

Rats were anesthetized with isoflurane and subjected to BOP using an ABS located at the Walter Reed Army Institute of Research (WRAIR). The ABS consists of a 0.5 ft long compression chamber that is separated from a 21 × 2 ft long expansion chamber that extends 3.6 ft into the end wave eliminator, which eliminates secondary shock wave long end wave eliminator (Sajja et al., 2018). The anesthetized rat was secured in the test section in a transverse (side-on; off-axis) orientation to the direction of BOP exposure. The compression chamber was pressurized with room air, causing membranes to rupture at a pressure that is dependent upon the thickness of the specific membrane sheet separating the two chambers, yielding a supersonic blast wave (shockwave) that impacts the experimental subject in the test section. The pressure data was recorded at 800,000 samples/s that is baselined to ambient pressure with TMX 18 data recorder (Astronova, West Warwick, RI, USA). To avoid poly-organ injury in these experiments, acetate membranes (Grafix Inc., Ohio, OH, USA) were used to yield peak positive static pressures of 8.5 psig (positive pressure-impulse: 11.46 psig*ms, duration: 2.94 ms; negative pressure-impulse: −8.35 psig*ms, peak: −2.86 psig, duration: 5.89 ms) or 10 psig (positive pressure-impulse: 16.64 psig*ms, duration: 4.0 ms; negative pressure-impulse: −10.27 psig*ms, peak: −2.34 psig, duration: 7.07 ms) with a positive phase duration of 3–5 ms) as in Sajja et al. (2020). Pressure profiles are shown in Figure 1. Animals (*n* = 6 per group) were exposed to a daily BOP of 8.5 or 10 psi 14 times (14×) from the side; repeated BOP exposures were separated by 24 h. All sham animals were subjected to isoflurane anesthesia, loading in the shock tube, and recovery procedures, but were not exposed to the BOP. At 24 h following final BOP exposure, animals were euthanized and whole hemisphere brain tissue was flash-frozen on dry-ice until further analysis.

### Protein Extraction

After euthanasia, the total soluble protein was extracted from the right cerebrum as described previously (Heyburn et al., 2019a). Briefly, the cerebrum was homogenized on ice in tissue protein extraction reagent (T-PER) with protease/phosphatase inhibitors and the soluble portion was separated for use in biochemical assays and stored at 𢀒80°C.

### Western Blot

Western blot samples were prepared and separated in NuPAGE^TM^ 4–12% 1.0 mm, 12-well Bis-Tris Protein Gels (cat# NP0322BOX, Thermo Fisher Scientific) as described previously (Heyburn et al., 2019a). Membranes were stripped and re-probed with a second antibody, and then re-probed with an antibody against β-actin to serve as a loading control. The proteins that were stripped and re-probed on the same membrane are Claudin-5 and occludin; TDP-43 and Piezo2; and Aβ40 and Aβ42. Bands were analyzed by densitometry analysis using ImageJ software (NIH) and protein levels were determined using β-actin as a loading control. Primary antibodies: rabbit polyclonal antibody against TDP-43 (1:2,000, ProteinTech cat# 10782-2-AP), rabbit polyclonal antibody against FAM38B/Piezo2 (1:2,000, ProSci cat# 26-438), mouse monoclonal antibody against Occludin (1:2,000, Thermo Fisher Scientific cat# 33-1500), mouse monoclonal antibody against Claudin-5 (1:2,000, Thermo Fisher Scientific cat# 35-2500), rabbit polyclonal antibody against amyloidβ-40 (1:2,000, Abcam cat# ab110888), rabbit polyclonal antibody against amyloidβ-42 (1:4,000, Abcam cat# ab10148), rabbit monoclonal antibody against pS396 Tau (1:1,333, Abcam cat# ab109390), and mouse monoclonal antibody against β-actin (1:20,000 Abcam A2228). Secondary antibodies: horseradish peroxidase-conjugated secondary antibodies against rabbit (1:2,500, cat# 65-6120) and mouse (1:2,500, Thermo Fisher Scientific cat# 32430). Full blot images in Supplementary Figure 1.

### Automated Western Blot With Jess System

GFAP levels were measured in soluble brain hemisphere homogenates using the Jess system (Protein Simple, cat# 004-650) according to the manufacturer’s instructions. Samples were loaded into a 12–230 kDa Jess Separation Module with 25 capillary cartridges (Protein Simple, cat# SM-W004). The anti-rabbit NIR detection module (Protein Simple, cat# DM-008) was used to detect the rabbit polyclonal GFAP antibody (Abcam, cat# ab7260). The anti-mouse IR detection module (Protein Simple, cat# DM-010) was used to detect the housekeeping protein β-actin (mouse monoclonal, Sigma–Aldrich, cat# a2228). The plate was centrifuged for 5 min at ~1000×g at room temperature, then read on the Jess module, with 30-min blocking time, 30-min separation time, and 18-s stacking time. The Jess software produced a curve of fluorescence signal for GFAP and β-actin. The area under the curve was measured, and GFAP was normalized to β-actin, and this was normalized to sham.

### Multiplex ELISA

Multiple cytokines and chemokine markers were measured in soluble brain hemisphere homogenates using Rat Magnetic Luminex Assay (R&D Systems, cat# LXSARM) according to the manufacturer’s instructions. All samples were run in triplicates according to the manufacturer’s instructions. Standards, quality controls, and samples were loaded into a 96-well plate. Magnetic beads corresponding to each cytokine/chemokine were added to the same wells and incubated for 2 h at room temperature. Plates were washed twice before the addition of detection antibodies, then incubated with agitation for 1 h at room temperature. Streptavidin–Phycoerythrin was added and incubated in the wells, which were then washed two times. Sheath fluid was added to all wells, and the plate was run in a Luminex MAGPIX with Luminex xPONENT 4.2 software. Analyte levels were calculated as protein concentration (pg/mg of total protein) based on a standard curve, then were normalized to sham. Analytes examined: Chemokine (C-X-C motif) ligand 2 (CXCL2), CXCL3, Intercellular Adhesion Molecule 1 (ICAM-1), interferon (IFN)γ, interleukin (IL)-1α, IL-1β, IL-2, IL-4, IL-6, IL-10, IL-18, tissue inhibitor of metalloproteinase (TIMP)1, TNF-α, and vascular endothelial growth factor (VEGF).

### Statistical Analysis

All data were normalized relative to sham levels. A one-way ANOVA test was performed, with Dunnett’s *post hoc* test, for each protein. A significance level of *p* < 0.05 was considered statistically significant. Unless otherwise specified, all data are expressed as mean ± SEM.

## Results

All the results reported are from samples collected 24 h following final BOP exposure.

### Inflammatory Response

**Figure 2:**
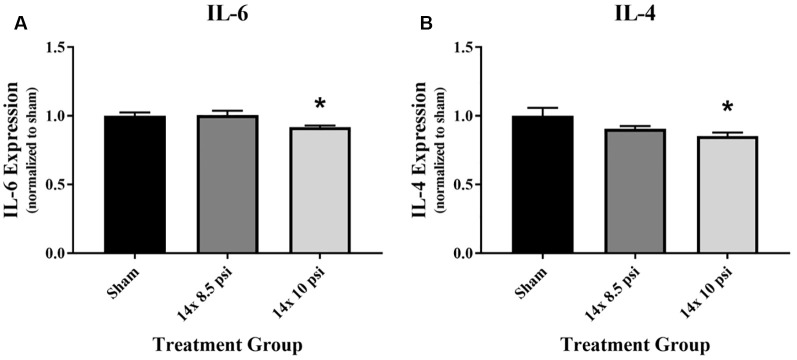
Cytokine alterations following repeated low-level blast overpressure (BOP) exposure. **(A)** Anti-inflammatory cytokine interleukin (IL)-4 was significantly reduced in the brain following 14 exposures to 10 psi overpressure. Sham average: 31.87 pg/mg; 14 × 8.5 psi average: 28.91 pg/mg; 14 × 10 psi average: 27.22 pg/mg of total protein. **(B)** Pro-inflammatory cytokine IL-6 was significantly reduced in the brain following 14 exposures to 10 psi overpressure. Sham average: 1,084.12 pg/mg; 14 × 8.5 psi average: 1,091.89 pg/mg; 14 × 10 psi average: 995.82 pg/mg of total protein. *p < 0.05.

An immunology multiplex assay was performed to measure 14 different markers: CXCL2, CXCL3, ICAM-1, IFNγ, IL-1α, IL-1β, IL-2, IL-4, IL-6, IL-10, IL-18, TIMP1, TNFα, and VEGF. Most of these molecules did not have any significant alteration after 14 exposures to either 8.5 or 10 psig BOP (Supplementary Figure 1), though the majority showed a trend of reduced levels. The anti-inflammatory cytokine IL-4 was significantly reduced (*sham* = 31.87 pg/mg of total protein, 14 × 10 psi = 27.22 pg/mg of total protein, ~15%) in the brain following repeated exposure to 10 psig overpressure (Figure 2A). The pro-inflammatory cytokine IL-6 was also significantly reduced (*sham* = 1084.12 pg/mg of total protein, 14 × 10 psi = 995.82 pg/mg of total protein, ~8%) in the brain following repeated 10 psi exposure (Figure 2B).

### BBB Proteins

**Figure 3:**
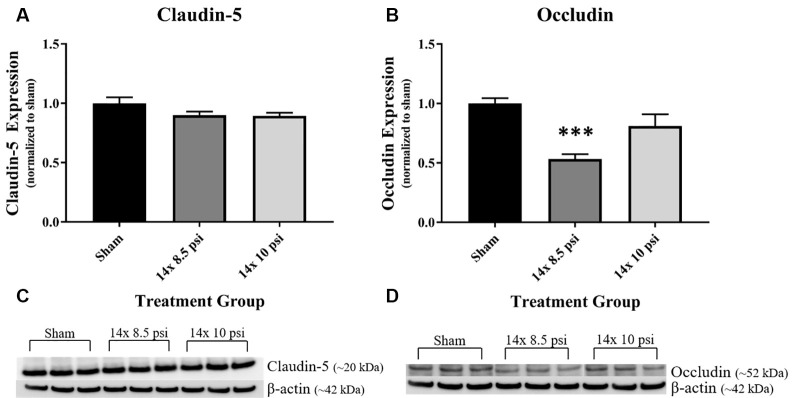
Tight junction proteins were reduced following repeated low-level BOP exposure. **(A)** The tight junction protein claudin-5 was reduced (*p* = 0.11) in the brain following 14 exposures to low-level BOP. **(B)** Representative Western blot image of claudin-5 and β-actin. **(C)** Occludin was significantly reduced in the brain following 14 exposures to 8.5 psi overpressure. **(D)** Representative Western blot image of occludin and β-actin. ***p < 0.001.

**Figure 4:**
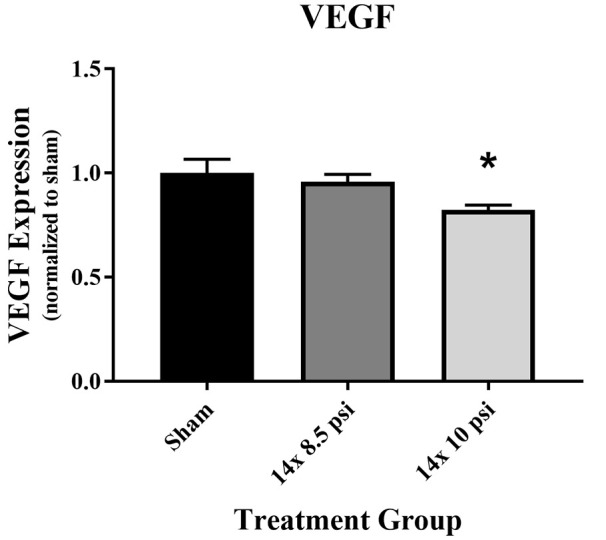
Vascular endothelial growth factor (VEGF) was reduced following repeated low-level BOP exposure. VEGF levels were significantly reduced in the brain following 14 exposures to 10 psi overpressure. Sham average: 165.19 pg/mg; 14 × 8.5 psi average: 158.18 pg/mg; 14 × 10 psi average: 136.14 pg/mg of total protein, *p < 0.05.

**Figure 5:**
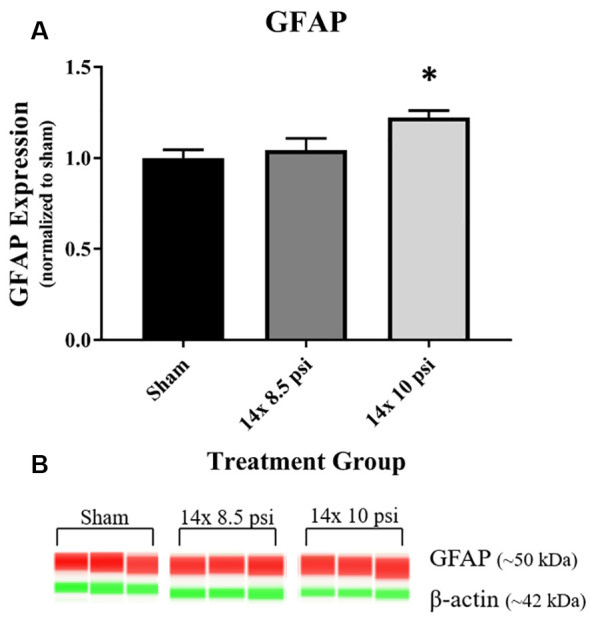
Astrocyte marker GFAP was increased following repeated low-level BOP exposure. **(A)** GFAP was significantly increased in the brain following 14 exposures to 10 psi overpressure. **(B)** Representative automated Western blot image of GFAP and β-actin. *p < 0.05.

Constituents underlying BBB integrity were assessed by measuring two tight junction proteins using Western blot. While not statistically significant, the levels of claudin-5 trended downward following repeated low-level BOP exposure (Figures 3A–C). Occludin was significantly reduced (~47%) in the brain following 14 exposures to 8.5 psi BOP, but there were no significant changes following 14 exposure to 10 psi BOP (Figures 3B–D). The angiogenesis-promoting protein VEGF was significantly reduced (~18%) in the brain following repeated exposure to 10 psi overpressure (Figure 4). GFAP, a marker of astrogliosis and a protein expressed in BBB astrocyte endfeet, was significantly increased (~22%) in the brain following 14 exposures to 10 psi BOP (Figure 5).

### Mechanosensitive Protein (Piezo2)

**Figure 6:**
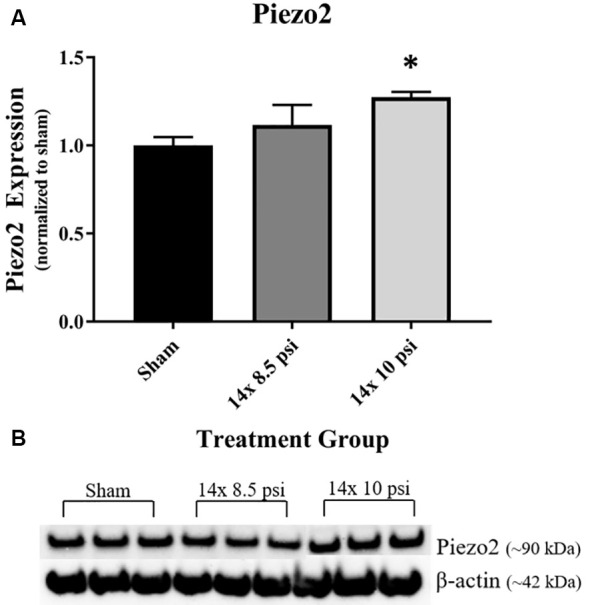
Mechanosensitive ion channel protein Piezo2 was altered following repeated low-level BOP exposure. **(A)** Piezo2 was significantly increased in the brain following 14 exposures to 10 psi overpressure. **(B)** Representative Western blot image of Piezo2 and β-actin. *p < 0.05.

Response to pressure changes in the brain as reflected by altered levels of the mechanosensitive ion channel protein, Piezo2. Piezo2 was significantly increased in the brain following repeated exposure to 10 psi overpressure, but there was no significant increase following 14 exposures to 8.5 psi BOP (Figure 6).

### Neurodegeneration-Related Proteins

**Figure 7:**
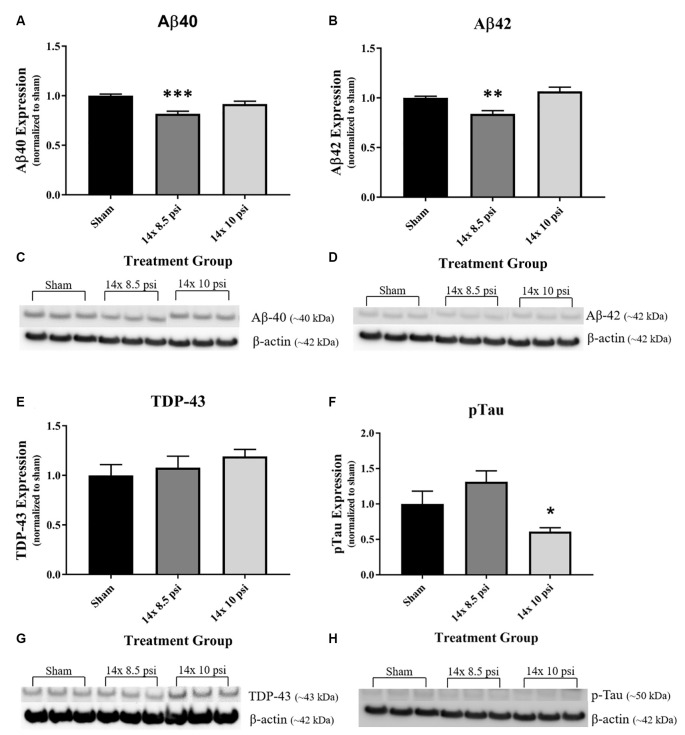
Alterations in neurodegeneration-related proteins following repeated low-level BOP exposure. **(A,B)** Aβ40 and Aβ42 were significantly reduced in the brain following 14 exposures to 8.5 psi overpressure. **(C,D)** Representative Western blot image of Aβ40, Aβ42, and β-actin. **(E)** TDP-43 was trending to increase in the brain following repeated low-level BOP exposure. **(F)** Representative Western blot image of TDP-43 and β-actin. **(G)** Phospho-S396 Tau was significantly reduced following 14 exposures to 10 psi overpressure. **(H)** Representative Western blot images of pTau and β-actin. *p < 0.05, **p < 0.01, ***p < 0.001.

Amyloid-β 40 and 42 (Aβ40, Aβ42), two proteins recognized in neurodegenerative disease, primarily Alzheimer’s disease (AD), were significantly reduced (~18% and ~16%, respectively) in the brain following 14 exposures to 8.5 psi BOP (Figures 7A–D). TDP-43, a protein with abnormal expression in several neurodegenerative diseases, was increased, though not significantly, in the brain following repeated low-level BOP exposure (Figures 7E–G). Tau phosphorylated at S396, a feature of Alzheimer’s disease-related tau pathology, was significantly decreased (~39%) following 14 exposures to 10 psi BOP (Figures 7F–H).

## Discussion

A variety of acute changes in the brain that relate to processes of brain injury and neurodegeneration were observed following repeated low-level BOP exposure. The BBB unit is an especially important focus here and the observed changes in levels of tight junction proteins, mediators of angiogenesis (VEGF), and GFAP-positive astrocytes could help in our understanding of the injury risk of BOP to the brain. Piezo2, a mechanosensitive ion channel protein, which was shown to actively support cerebrovascular integrity by regulation of endothelial cells and angiogenesis could similarly play an important role in our understanding of blast pathophysiology (Ferrari et al., 2015; Yang et al., 2016; Zhang et al., 2017; Heyburn et al., 2019a). Piezo2 leads to cation channel activation and signal transduction in neurons and other cell types (Coste et al., 2010), and therefore we predict that increases in Piezo2 expression may cause hypersensitivity of cells in the brain in the response to pressure changes, leading to altered signal transduction. Daily exposure to either 8.5 or 10 psi BOP was shown to produce alterations in regulators of the BBB, reduction in levels of inflammatory markers, and decreased levels of neurodegeneration-related proteins. Additionally, increased levels of astrocyte-specific GFAP and increased pressure sensitivity (Piezo2) in the brain was observed. Together, these data indicate that repeated daily exposure to low-level BOP has significant effects on the brain in the short term, and these effects may contribute to long-term deficits reported by Warfighters. Importantly, the low intensities of BOP studied here do not cause gross lung injury (Sajja et al., 2020), so these effects are likely due to the direct effects of BOP on the brain rather than as the indirect result of systemic injuries.

Inflammation is a major component of TBI and likely plays a role in the pathogenesis of blast-related brain injury (Readnower et al., 2010; Corps et al., 2015). In this study, two cytokines (IL-4 and IL-6) were significantly altered after repeated low-level BOP exposure. Though many studies of BOP exposure using animal models have demonstrated an increase in pro-inflammatory cytokines such as IL-1β, IL-6, IL-8, and TNFα in the brain (Kovesdi et al., 2011; Cho et al., 2013; Perez-Polo et al., 2014; Elder et al., 2015), most of the inflammatory markers we measured were reduced, though not significantly, compared to shams (Supplementary Figure 1). These differences across studies might be attributed to higher exposure pressures along with experimental methodologies, specifically the use of cylindrical blast simulators in the previous studies, which impart high dynamic pressure (i.e., blast wind), plateaued peaks, and long duration blast waves (Sajja et al., 2018). In the current study, the anti-inflammatory cytokine IL-4 and pro-inflammatory cytokine IL-6 were significantly reduced in the brain following repeated exposure to 10 psi BOP (Figure 2), whereas Gill et al. (2017a, 2017b) found increased peripheral IL-6 in blood samples of human subjects participating in breaching training, though this increase could be a contribution from peripheral organs affected by the BOP exposure (Gill et al., 2017a). In addition to perhaps reflecting bidirectional changes in the brain and the circulation, it is also possible that brain changes are limited and restricted to discrete neuroanatomical locations that were obscured in our larger tissue measurements of the whole cerebrum. On the other hand, a recent study comparing pre-and post-deployment peripheral cytokine levels in populations experiencing mild bTBI showed decreased levels of IL-4 and IL-6 after deployment, which is a finding seemingly more consistent with our observations in the current study (Rusiecki et al., 2020). Overall reductions in inflammatory markers, whether pro- or anti-inflammatory, indicates that there is no strong immune response following repeated low-level BOP, contrary to what might be expected following more severe injury. These molecules, along with other proteins, may also have been cleared from the brain *via* the glymphatic system, which is responsible for clearing the brain of proteins (Iliff et al., 2012). The differences between the two intensity groups may be due to differing time course of pathogenesis, or involvement of different signaling cascades due to poly-organ injury following 14 × 10 psi BOP exposure.

Breakdown of the BBB, which is a common outcome of BOP exposure in animal models, is likely a pivotal contributor to the pathophysiology of BOP insults to the brain (Readnower et al., 2010; Yeoh et al., 2013; Shetty et al., 2014; Hue et al., 2016; Kawoos et al., 2016). We previously reported disruption in tight junction proteins and VEGF following higher intensity BOP exposure (Heyburn et al., 2019a). In the current study, we found that VEGF and the tight junction proteins claudin-5 and occludin are decreased following repeated low-level BOP exposure, with a significant reduction in occludin after repeated 8.5 psi BOP and a significant reduction in VEGF after repeated 10 psi BOP exposure (Figure 3). Similar disruptions in tight junction proteins have been reported in rats following higher intensity BOP exposure (Abdul-Muneer et al., 2013) and in humans undergoing heavy weapons training (Duckworth, 2018), indicating that BBB disruption may be a common effect of BOP exposure across a range of intensities. Though we observed a reduction in VEGF levels, presumably indicating an absence of angiogenesis, other groups have found that BOP exposure leads to elevated VEGF levels (Sajja et al., 2012a; Kamnaksh et al., 2014; Hubbard et al., 2017; Gama Sosa et al., 2017). It is important to note that these studies used higher intensity BOP than was used in this study, which could have resulted in poly-organ injury. Overall, tight junction proteins appear to be vulnerable to BOP at varying intensities, whereas VEGF was increased following higher intensity BOP (Heyburn et al., 2019a) but decreased following repeated low-intensity BOP (Figure 4), a result that could help distinguish among pathophysiological responses to BOP.

GFAP, an astrocyte-specific protein that is increased during astrogliosis in response to injury, has been recognized as a serum biomarker to predict mild-to-moderate TBI, astrocyte injury, and BBB damage (Papa et al., 2012; Eonta et al., 2019). Astrocytes are involved in inflammatory and repair processes in the brain, and are also a major component of the BBB, with astrocytic end feet encircling blood vessels in the brain microvasculature and assisting in the maintenance of tight junction integrity (Shetty et al., 2014). In this study, we found that GFAP is significantly increased in the brain following exposure to repeated 10 psi BOP (Figure 5). The measured increase in GFAP may be part of a compensatory or regenerative mechanism elicited to repair the damaged BBB by increasing or restoring astrocytic endfeet at the blood vessels. To determine the cause or consequence of the increase in GFAP, future experiments employing immunohistochemical analysis will be required to distinguish the neuroanatomical localization of GFAP responses in the brain. It is speculated that both inflammatory and BBB restoration mechanisms are activated in tandem to protect the brain against cellular loss and to maintain cellular homeostasis.

It has been shown that BOP can cause large ICP changes, and the degree of response to these changes in pressure is speculated to correlate with the severity of the injury, such that altered mechanosensitivity in the brain could make it more vulnerable to injury from subsequent BOP exposure (Coste et al., 2010; Sajja et al., 2018). Piezo2, when activated by pressure changes such as BOP, leads to mechanotransduction, which may play a role in cellular injury response to brain injury (Hemphill et al., 2015; Szczot et al., 2017; Shin et al., 2019). Our group previously reported that Piezo2 is increased in the brain after high-intensity BOP exposure and that its levels correlated with BOP intensity (Heyburn et al., 2019a). In the current study, we again observed that Piezo2 is significantly increased in the brain following repeated exposures to 10 psi, but not 8.5 psi, BOP. Together, these findings indicate that high intensity and repeated low-intensity BOP exposure lead to increased levels of Piezo2, which may, in turn, lead to increased sensitivity to subsequent changes in pressure in the brain. Also, Piezo2 is involved in vascular endothelial cell-dependent mechanotransduction (Ferrari et al., 2015), pointing to a role for Piezo2 in vascular regulation that could contribute to BBB homeostasis. The increased Piezo2 following 10 psi BOP exposures complements the observed changes in GFAP and could potentially reflect roles for each in BBB structure and function.

Increasingly, TBI has been associated with neurodegenerative processes and neurodegeneration-related protein abnormalities (DeKosky and Asken, 2017; Heyburn et al., 2019b). Although significant alterations in brain levels of TDP-43, a marker associated with neurodegenerative disease processes, were previously observed following moderate-intensity BOP exposure (Heyburn et al., 2019a), we did not observe a significant change in TDP-43 levels following repeated low-level BOP exposure in the current study, though it was trending upward with increased BOP intensity (Figure 6). We observed significant reductions in Alzheimer’s disease-related proteins following repeated low-level BOP, with phosphorylated tau (S396) significantly reduced after repeated 10 psi BOP exposure, and Aβ40 and Aβ42 significantly reduced after repeated 8.5 psi BOP exposure (Figure 7). Similarly, a reduction in Aβ42 was observed in human serum samples 24 h after exposure to BOP during breaching training (Edwards et al., 2020). A study of Warfighters in breaching training found that amyloid precursor protein (APP), whose cleavage products include Aβ40 and Aβ42, is reduced in the blood following training related BOP exposure at acute time points (Gill et al., 2017b), which aligns with our findings of reduced Aβ40 and Aβ42 following repeated low-level BOP exposure. In a case study of a Warfighter who sustained three BOP exposures, the brain was positive for Aβ plaques and phosphorylated tau lesions, but negative for TDP-43 inclusions (Iacono et al., 2020). While this clinical finding fits with our TDP-43 data in this study, it is inconsistent with our Aβ40, Aβ42, and pTau data (Iacono et al., 2020), which could be attributed to the severity of BOP and the chronic nature of outcomes in the clinical study. The early changes we observed in Aβ and pTau could eventually lead to an increase in these markers at a chronic time-point, which needs to be further investigated in future studies.

For several of the proteins studied (occludin, Aβ40, Aβ42), there were significant alterations in the 14 × 8.5 psi BOP group, but not in the higher intensity (14 × 10 psi) group. Similar findings were reported in a study with single low-level BOP exposure (De Gasperi et al., 2012). It was observed that Aβ40 and Aβ42 were decreased at lower pressures (either 5 or 10 psi) but not in the 17 psi group (De Gasperi et al., 2012). The higher intensity exposure may cause poly-organ injury, rather than just primary BOP injury to the brain, leading to divergent downstream cellular and molecular events. Another explanation is that at lower intensities, there is enhanced glymphatic flow, which involves clearance of molecules from the brain parenchyma *via* aquaporin-4 channels in astrocytes (Iliff et al., 2012). There have been studies of blast overpressure showing that AQP4 is increased following blast exposure (Gu et al., 2017; Rios et al., 2019). It is speculated that mechanisms of glymphatic flow could be associated with the decrease in some proteins and cytokines following BOP exposure. It is also possible that following a single BOP exposure, the brain is primed for subsequent blast exposure, which may result in immune tolerance and activation, which needs to be further investigated.

## Conclusion

In this study, we identified several pathophysiological responses to repeated low-level BOP exposure, similar to that experienced by Warfighters in training environments. These exposures lead to acute changes in the brain including disruption of protein regulators of the BBB, astrogliosis, increased levels of a mechanosensitive protein, a decrease in brain cytokines, and decreased neurodegeneration-related proteins, outcomes which reflect overall perturbation of the brain by BOP forces. These changes observed in this study could potentially contribute to cumulative effects of repeated BOP exposure that could, in turn, contribute to the symptoms and behavioral deficits experienced by Warfighters. Eventually, these data can help contribute to the development of brain injury risk curves for low-level BOP and ultimately lead to the development of protective standards to prevent acute and chronic blast effects.

## Data Availability Statement

The raw data supporting the conclusions of this article will be made available by the authors, without undue reservation.

## Ethics Statement

The animal study was reviewed and approved by Walter Reed Army Institute of Research IACUC.

## Author Contributions

LH and VS designed the experiments. LH wrote the manuscript. DW performed the blast experiments. LH, RA, SG, AB, RU, JW, and PA performed the experimental procedures. LH and RA performed the data analysis. LH performed the statistical analysis. JL, SA, and VS oversaw the study. All authors contributed to the article and approved the submitted version.

## Disclaimer

Material has been reviewed by the Walter Reed Army Institute of Research. There is no objection to its presentation and/or publication. The opinions or assertions contained herein are the private views of the authors and are not to be construed as official, or as reflecting true views of the Department of the Army, Department of the Navy, or the Department of Defense. The research was conducted under an approved animal use protocol in an AAALACi accredited facility in compliance with the Animal Welfare Act and other federal statutes and regulations relating to animals and experiments involving animals and adheres to principles stated in the Guide for the Care and Use of Laboratory.

JL and SA are employees of the U.S. Government. This work was prepared as part of their official duties. Title 17, U.S.C., §105 provides that copyright protection under this title is not available for any work of the U.S. Government. Title 17, U.S.C., §101 defines a U.S. Government work as a work prepared by an employee of the U.S. Government as part of that person’s official duties.

## Conflict of Interest

The authors declare that the research was conducted in the absence of any commercial or financial relationships that could be construed as a potential conflict of interest.

## Supplementary Material

The Supplementary Material for this article can be found online at: https://www.frontiersin.org/articles/10.3389/fncel.2021.636707/full# supplementary-material.

## References

[B1] Abdul-Muneer P. M. Schuetz H. Wang F. Skotak M. Jones J. Gorantla S.. (2013). Induction of oxidative and nitrosative damage leads to cerebrovascular inflammation in an animal model of mild traumatic brain injury induced by primary blast. Free Radic. Biol. Med. 60, 282–291. 10.1016/j.freeradbiomed.2013.02.029 23466554 PMC4007171

[B2] Bolander R. Mathie B. Bir C. Ritzel D. VandeVord P. (2011). Skull flexure as a contributing factor in the mechanism of injury in the rat when exposed to a shock wave. Ann. Biomed. Eng. 39, 2550–2559. 10.1007/s10439-011-0343-0 21735320

[B3] Carr W. Polejaeva E. Grome A. Crandall B. LaValle C. Eonta S. E.. (2015). Relation of repeated low-level blast exposure with symptomology similar to concussion. J. Head Trauma Rehabil. 30, 47–55. 10.1097/HTR.0000000000000064 24901327

[B4] Carr W. Stone J. R. Walilko T. Young L. A. Snook T. L. Paggi M. E.. (2016). Repeated low-level blast exposure: a descriptive human subjects study. Mil. Med. 181, 28–39. 10.7205/MILMED-D-15-00137 27168550

[B5] Cernak I. Merkle A. C. Koliatsos V. E. Bilik J. M. Luong Q. T. Mahota T. M.. (2011). The pathobiology of blast injuries and blast-induced neurotrauma as identified using a new experimental model of injury in mice. Neurobiol. Dis. 41, 538–551. 10.1016/j.nbd.2010.10.025 21074615

[B6] Cho H. J. Sajja V. S. S. S. Vandevord P. J. Lee Y. W. (2013). Blast induces oxidative stress, inflammation, neuronal loss and subsequent short-term memory impairment in rats. Neuroscience 253, 9–20. 10.1016/j.neuroscience.2013.08.037 23999126

[B7] Corps K. N. Roth T. L. McGavern D. B. (2015). Inflammation and neuroprotection in traumatic brain injury. JAMA Neurol. 72, 355–362. 10.1001/jamaneurol.2014.3558 25599342 PMC5001842

[B8] Coste B. Mathur J. Schmidt M. Earley T. J. Ranade S. Petrius M. J.. (2010). Piezo1 and Piezo2 are essential components of distinct mechanically activated cation channels. Science 330, 55–60. 10.1126/science.1193270 20813920 PMC3062430

[B9] De Gasperi R. Gama Sosa M. A. Kim S. H. Steele J. W. Shaughness M. C. Maudlin-Jeronimo E.. (2012). Acute blast injury reduced brain abeta in two rodent species. Front. Neurol. 3:177. 10.3389/fneur.2012.00177 23267342 PMC3527696

[B10] DeKosky S. T. Asken B. M. (2017). Injury cascades in TBI-related neurodegeneration. Brain Inj. 31, 1177–1182. 10.1080/02699052.2017.1312528 28981345 PMC6218169

[B11] DeKosky S. T. Ikonomovic M. D. Gandy S. (2010). Traumatic brain injury—football, warfare, and long-term effects. N. Engl. J. Med. 363, 1293–1296. 10.1056/NEJMp1007051 20879875

[B12] Duckworth J. (2018). “Understanding potential neurological consequences and mechanisms of repeated blast exposure,” in Seventh State-of-the-Science Meeting, The Neurological Effects of Repeat Exposure to Military Occupational Blast: Implications for Prevention and Health (Arlington, VA), 15–18.

[B13] Edwards K. A. Leete J. J. Tschiffely A. E. Moore C. Y. Dell K. C. Statz J. K.. (2020). Blast exposure results in tau and neurofilament light chain changes in peripheral blood. Brain Inj. 34, 1213–1221. 10.1080/02699052.2020.1797171 32755419

[B14] Elder G. A. Gama Sosa M. A. De Gasperi R. Radford Stone J. Dickstein D. L. Haghighi F.. (2015). Vascular and inflammatory factors in the pathophysiology of blast-induced brain injury. Front. Neurol. 6:48. 10.3389/fneur.2015.00048 25852632 PMC4360816

[B15] Eonta S. E. Kamimori G. H. Wang K. K. W. Carr W. LaValle C. R. Egnoto M. J.. (2019). Case study of a breacher: investigation of neurotrauma biomarker levels, self-reported symptoms, and functional MRI analysis before and after exposure to measured low-level blast. Mil. Med. 185, e513–e517. 10.1093/milmed/usz185 31429467

[B16] Ferrari L. F. Bogen O. Green P. Levine J. D. (2015). Contribution of Piezo2 to endothelium-dependent pain. Mol. Pain 11:65. 10.1186/s12990-015-0068-4 26497944 PMC4619430

[B17] Gama Sosa M. A. De Gasperi R. Perez Garcia G. S. Sosa H. Searcy C. Vargas D.. (2017). Lack of chronic neuroinflammation in the absence of focal hemorrhage in a rat model of low-energy blast-induced TBI. Acta Neuropathol. Commun. 5:80. 10.1186/s40478-017-0483-z 29126430 PMC6389215

[B18] Gill J. Cashion A. Osier N. Arcurio L. Motamedi V. Dell K. C.. (2017a). Moderate blast exposure alters gene expression and levels of amyloid precursor protein. Neurol. Genet. 3:e186. 10.1212/NXG.0000000000000186 28975156 PMC5618107

[B19] Gill J. Motamedi V. Osier N. Dell K. Arcurio L. Carr W.. (2017b). Moderate blast exposure results in increased IL-6 and TNFα in peripheral blood. Brain Behav. Immun. 65, 90–94. 10.1016/j.bbi.2017.02.015 28232173 PMC5537025

[B20] Gu M. Kawoos U. McCarron R. Chavko M. (2017). Protection against blast-induced traumatic brain injury by increase in brain volume. Biomed. Res. Int. 2017:2075463. 10.1155/2017/2075463 28553646 PMC5434276

[B21] Hemphill M. A. Dauth S. Yu C. J. Dabiri B. E. Parker K. K. (2015). Traumatic brain injury and the neuronal microenvironment: a potential role for neuropathological mechanotransduction. Neuron 85, 1177–1192. 10.1016/j.neuron.2015.02.041 25789754

[B22] Heyburn L. Abutarboush R. Goodrich S. Urioste R. Batuure A. Statz J.. (2019a). Repeated low-level blast overpressure leads to endovascular disruption and alterations in TDP-43 and Piezo2 in a rat model of blast TBI. Front. Neurol. 10:766. 10.3389/fneur.2019.00766 31417481 PMC6682625

[B23] Heyburn L. Sajja V. S. S. S. Long J. B. (2019b). The role of TDP-43 in military-relevant TBI and chronic neurodegeneration. Front. Neurol. 10:680. 10.3389/fneur.2019.00680 31316455 PMC6610302

[B24] Hubbard W. B. Greenberg S. Norris C. Eck J. Lavik E. VandeVord P. (2017). Distinguishing the unique neuropathological profile of blast polytrauma. Oxid. Med. Cell. Longev. 2017:5175249. 10.1155/2017/5175249 28424745 PMC5382305

[B25] Hue C. D. Cho F. S. Cao S. Nicholls R. E. Vogel E. W. III. Sibindi C.. (2016). Time course and size of blood-brain barrier opening in a mouse model of blast-induced traumatic brain injury. J. Neurotrauma 33, 1202–1211. 10.1089/neu.2015.4067 26414212

[B26] Iacono D. Lee P. Edlow B. L. Gray N. Fischl B. Kenney K.. (2020). Early-onset dementia in war veterans: brain polypathology and clinicopathologic complexity. J. Neuropathol. Exp. Neurol. 79, 144–162. 10.1093/jnen/nlz122 31851313 PMC6970453

[B27] Iliff J. J. Wang M. Liao Y. Plogg B. A. Peng W. Gundersen G. A.. (2012). A paravascular pathway facilitates CSF flow through the brain parenchyma and the clearance of interstitial solutes, including amyloid β. Sci. Transl. Med. 4:147ra111. 10.1126/scitranslmed.3003748 22896675 PMC3551275

[B28] Kamimori G. H. LaValle C. R. Eonta S. Carr W. Tate C. Want K. K. W. (2018). Longitudinal investigation of neurotrauma serum biomarkers, behavioral characterization, and brain imaging in soldiers following repeated low-level blast exposure (New Zealand breacher study). Mil. Med. 183, 28–33. 10.1093/milmed/usx186 29635591

[B29] Kamnaksh A. Ahmed F. Kovesdi E. Barry E. S. Grunberg N. E. Long J. B.. (2014). Molecular mechanisms of increased cerebral vulnerability after repeated mild blast-induced traumatic brain injury. Transl. Proteom. 3, 22–37. 10.1016/j.trprot.2013.11.001

[B30] Kawoos U. Gu M. Lankasky J. McCarron R. M. Chavko M. (2016). Effects of exposure to blast overpressure on intracranial pressure and blood-brain barrier permeability in a rat model. PLoS One 11:e0167510. 10.1371/journal.pone.0167510 27907158 PMC5132256

[B31] Kovesdi E. Gyorgy A. B. Kwon S.-K. C. Wingo D. L. Kamnaksh A. Long J. B.. (2011). The effect of enriched environment on the outcome of traumatic brain injury; a behavioral, proteomics, and histological study. Front. Neurosci. 5:42. 10.3389/fnins.2011.00042 21503146 PMC3072528

[B32] Papa L. Lewis L. M. Falk J. L. Zhang Z. Silvestri S. Giordano P.. (2012). Elevated levels of serum glial fibrillary acidic protein breakdown products in mild and moderate traumatic brain injury are associated with intracranial lesions and neurosurgical intervention. Ann. Emerg. Med. 59, 471–483. 10.1016/j.annemergmed.2011.08.021 22071014 PMC3830977

[B33] Perez-Polo J. R. Rea H. C. Johnson K. M. Parsley M. A. Unabia G. C. Xu G.-Y.. (2014). A rodent model of mild traumatic brain blast injury. J. Neurosci. Res. 93, 549–561. 10.1002/jnr.23513 25410497

[B34] Readnower R. D. Chavko M. Adeeb S. Conroy M. D. Pauly J. R. McCarron R. M.. (2010). Increase in blood-brain barrier permeability, oxidative stress, and activated microglia in a rat model of blast-induced traumatic brain injury†. J. Neurosci. Res. 88, 3530–3539. 10.1002/jnr.22510 20882564 PMC2965798

[B35] Rios J. D. Choi J. H. McDaniel J. S. Becera S. Bice L. Johnson P.. (2019). Altered expression of aquaporin 1 and aquaporin 5 in the cornea after primary blast exposure. Mol. Vis. 25, 283–294. 31263351 PMC6571126

[B36] Rubovitch V. Ten-Bosch M. Zohar O. Harrison C. R. Tempel-Brami C. Stein E.. (2012). A mouse model of blast-induced mild traumatic brain injury. Exp. Neurol. 232, 280–289. 10.1016/j.expneurol.2011.09.018 21946269 PMC3202080

[B37] Rusiecki J. Levin L. I. Wang L. Byrne C. Krishnamurthy J. Chen L.. (2020). Blast traumatic brain injury and serum inflammatory cytokines: a repeated measures case-control study among U.S. military service members. J. Neuroinflammation 17:20. 10.1186/s12974-019-1624-z 31931830 PMC6958571

[B42] Sajja V. S. S. S. Arun P. Van Albert S. A. Long J. B. (2018). “Rodent model of primary blast-induced traumatic brain injury: guidelines to blast methodology,” in Pre-Clinical and Clinical Methods in Brain Trauma Research, Vol. 139, eds Cox C. Srivastava A. (New York, NY: Humana Press), 123–138.

[B41] Sajja V. S. S. S. Galloway M. P. Ghoddoussi F. Thiruthalinathan D. Kepsel A. Hay K.. (2012a). Blast-induced neurotrauma leads to neurochemical changes and neuronal degeneration in the rat hippocampus. NMR Biomed. 25, 1331–1339. 10.1002/nbm.2805 22549883

[B38] Sajja V. S. S. S. Tenn C. McLaws L. J. VandeVord P. J. (2012b). A temporal evaluation of cytokines in rats after blast exposure. Biomed. Sci. Instrum. 48, 374–379. 22846308

[B39] Sajja V. S. S. S. LaValle C. Salib J. E. Misistia A. C. Ghebremedhin M. Y. Ramos A. N.. (2019). The role of very low level blast overpressure in symptomatology. Front. Neurol. 10:891. 10.3389/fneur.2019.00891 31555194 PMC6722183

[B40] Sajja V. S. S. S. Statz J. K. Walker P. B. Gist I. D. Wilder D. M. Ahlers S. T.. (2020). Pulmonary injury risk curves and behavioral changes from blast overpressure exposures of varying frequency and intensity in rats. Sci. Rep. 10:16644. 10.1038/s41598-020-73643-7 33024181 PMC7538583

[B43] Schrenk-Siemens K. Wende H. Prato V. Song K. Rostock C. Loewer A.. (2014). Piezo2 is required for mechanotransduction in human stem cell-derived touch receptors. Nat. Neurosci. 18, 10–16. 10.1038/nn.3894 25469543

[B44] Shetty A. K. Mishra V. Kodali M. Hattiangady B. (2014). Blood brain barrier dysfunction and delayed neurological deficits in mild traumatic brain injury induced by blast shock waves. Front. Cell. Neurosci. 8:232. 10.3389/fncel.2014.00232 25165433 PMC4131244

[B45] Shin K. C. Park H. J. Kim J. G. Lee I. H. Cho H. Park C.. (2019). The Piezo2 ion channel is mechanically activated by low-threshold positive pressure. Sci. Rep. 9:6446. 10.1038/s41598-019-42492-4 31015490 PMC6478859

[B46] Shively S. B. Horkayne-Szakaly I. Jones R. V. Kelly J. P. Armstrong R. C. Perl D. P. (2016). Characterisation of interface astroglial scarring in the human brain after blast exposure: a post-mortem case series. Lancet Neurol. 15, 944–953. 10.1016/S1474-4422(16)30057-6 27291520

[B47] Simard J. M. Pampori A. Keledijan K. Tosun C. Schwartzbauer G. Ivanova S.. (2014). Exposure of the thorax to a sublethal blast wave causes a hydrodynamic pulse that leads to perivenular inflammation in the brain. J. Neurotrauma 31, 1292–1304. 10.1089/neu.2013.3016 24673157 PMC4108981

[B48] Szczot M. Pogorzala L. A. Solinski H. J. Young L. Yee P. Le Pinchon C. E.. (2017). Cell-type-specific splicing of Piezo2 regulates mechanotransduction. Cell Rep. 21, 2760–2771. 10.1016/j.celrep.2017.11.035 29212024 PMC5741189

[B49] Tate C. M. Wang K. K. W. Eonta S. Zhang Y. Carr W. Tortella F. C.. (2013). Serum brain biomarker level, neurocognitive performance, and self-reported symptom changes in soldiers repeatedly exposed to low-level blast: a breacher pilot study. J. Neurotrauma 30, 1620–1630. 10.1089/neu.2012.2683 23687938

[B50] Valiyaveettil M. Alamneh Y. Wang Y. Arun P. Oguntayo S. Wei Y.. (2013). Contribution of systemic factors in the pathophysiology of repeated blast-induced neurotrauma. Neurosci. Lett. 539, 1–6. 10.1016/j.neulet.2013.01.028 23370286

[B51] Yamamoto S. DeWitt D. S. Prough D. S. (2018). Impact and blast traumatic brain injury: implications for therapy. Molecules 23:245. 10.3390/molecules23020245 29373501 PMC6017013

[B52] Yang H. Liu C. Zhou R.-M. Yao J. Li X.-M. Shen Y.. (2016). Piezo2 protein: a novel regulator of tumor angiogenesis and hyperpermeability. Oncotarger 7, 44630–44643. 10.18632/oncotarget.10134 27329839 PMC5190124

[B53] Yeoh S. Bell E. D. Monson K. L. (2013). Distribution of blood-brain barrier disruption in primary blast injury. Ann. Biomed. Eng. 41, 2206–2214. 10.1007/s10439-013-0805-7 23568152

[B55] Zhang T. Chi S. Jiang F. Zhao Q. Xiao B. (2017). A protein interaction mechanism for suppressing the mechanosensitive Piezo channels. Nat. Commun. 8:1797. 10.1038/s41467-017-01712-z 29176668 PMC5702604

[B54] Zhang M. Wang Y. Geng J. Zhou S. Xiao B. (2019). Mechanically activated piezo channels mediate touch and suppress acute mechanical pain response in mice. Cell Rep. 26, 1419–1431. 10.1016/j.celrep.2019.01.056 30726728

[B56] Ziebell J. M. Morganti-Kossmann M. C. (2010). Involvement of pro- and anti-inflammatory cytokines and chemokines in the pathophysiology of traumatic brain injury. Neurotherapeutics 7, 22–30. 10.1016/j.nurt.2009.10.016 20129494 PMC5084109

