# Supplementary material for: Repeated Low-Level Blast Acutely Alters Brain Cytokines, Neurovascular Proteins, Mechanotransduction, and Neurodegenerative Markers in a Rat Model
Source: Front Cell Neurosci. 2021 Feb 19;15:636707. doi: 10.3389/fncel.2021.636707 (PMC7933446; doi:10.3389/fncel.2021.636707)
Supplement: Supplementary file 1 [file Data_Sheet_1.PDF]

Supplementary Material

1.1 Supplementary Figures

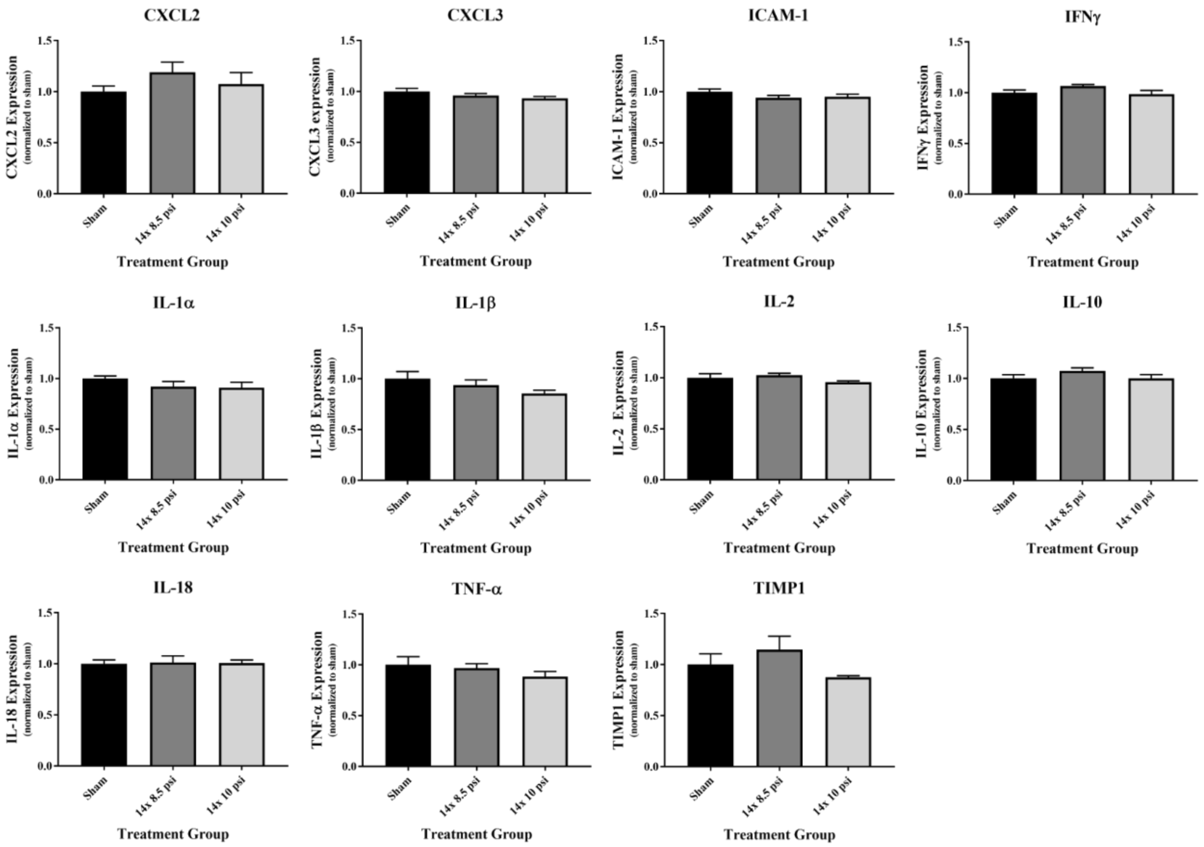

Supplementary Figure 1. Multiplex ELISA of multiple cytokine and chemokine markers measured in soluble brain hemisphere homogenates using Milliplex MAP Rat cytokine/chemokine magnetic bead panel (EMD Millipore RECYTMAG-65K).

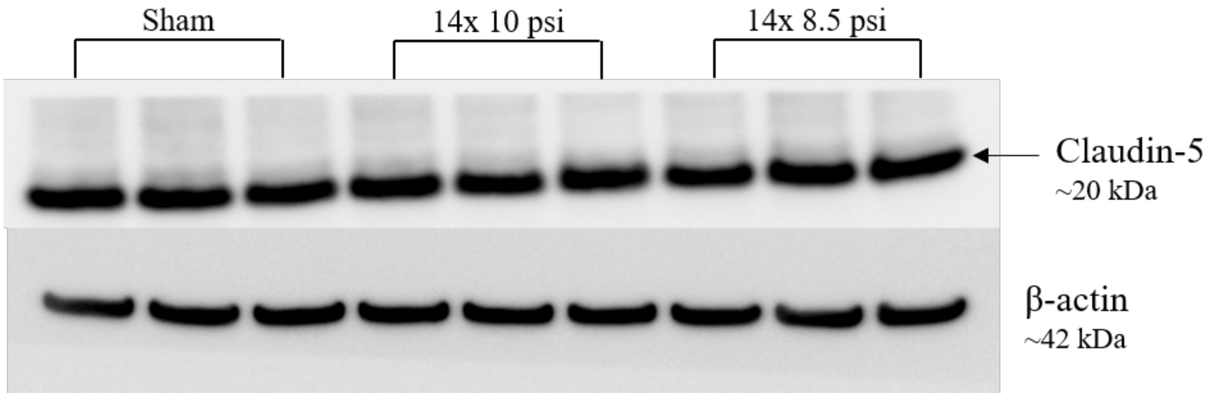

Supplementary Figure 2: One full Western blot of claudin-5 and β-actin.

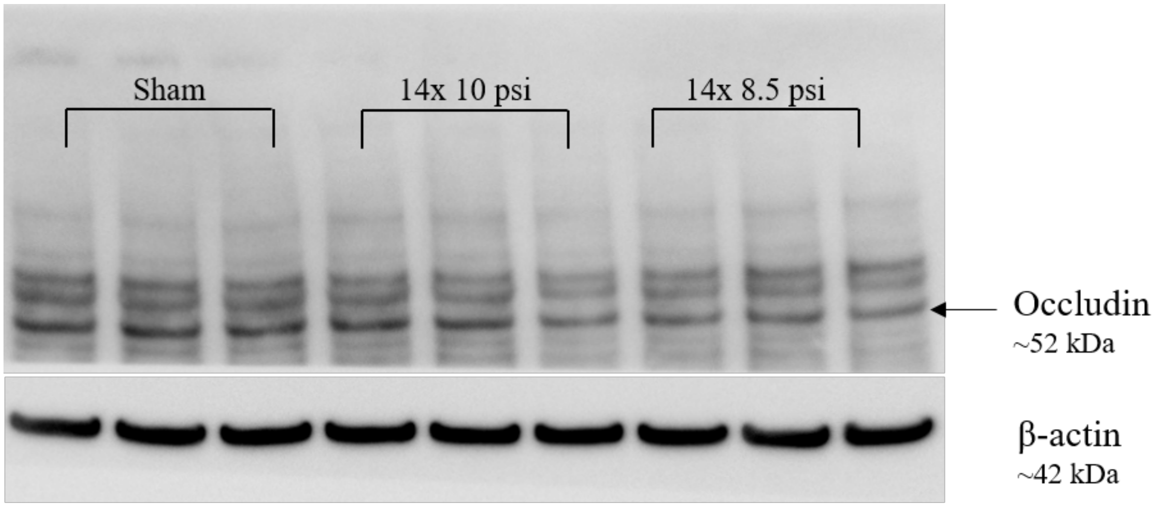

Supplementary Figure 3: One full Western blot of occludin and β-actin.

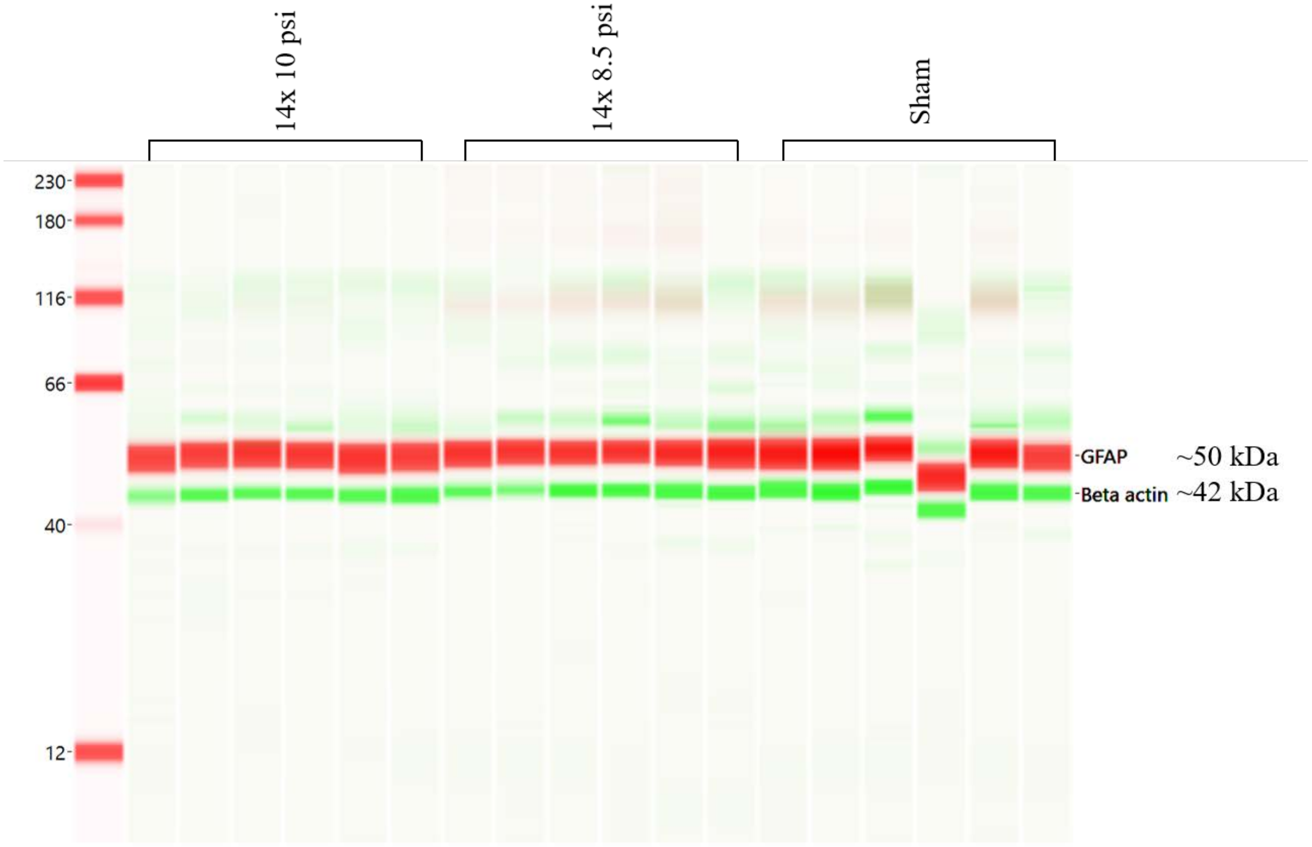

Supplementary Figure 4: One full Western blot of GFAP and β-actin.

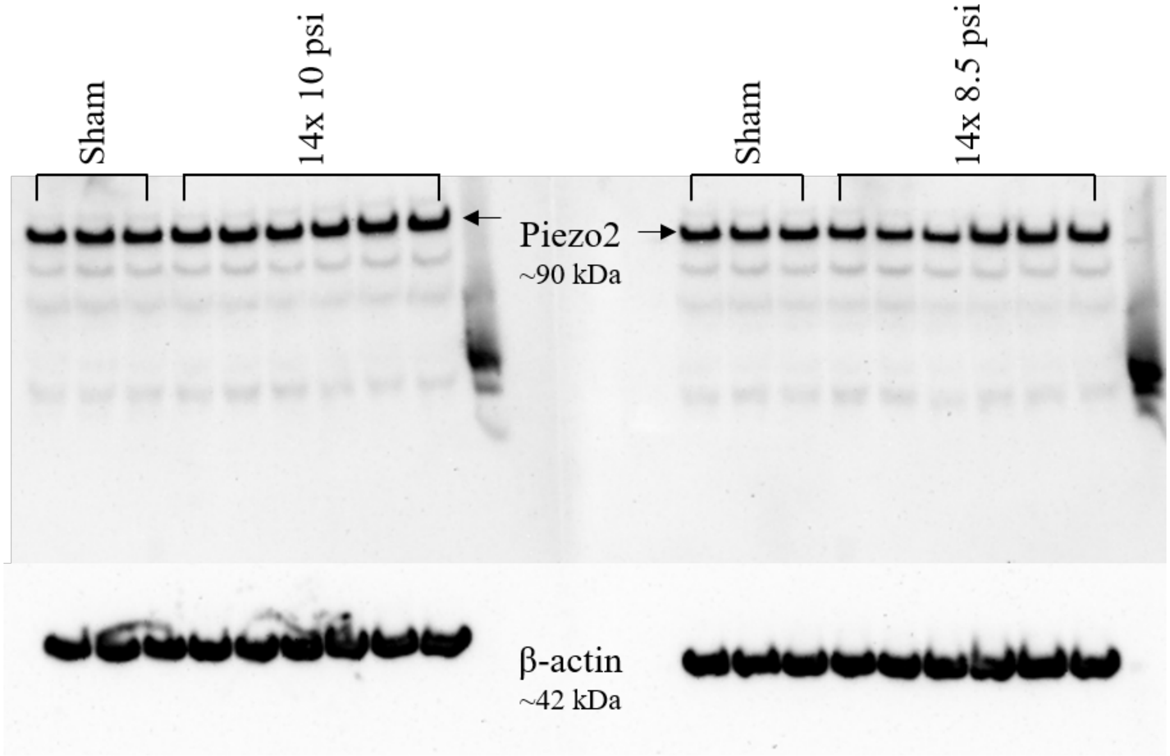

Supplementary Figure 5: One full Western blot of Piezo2 and β-actin.

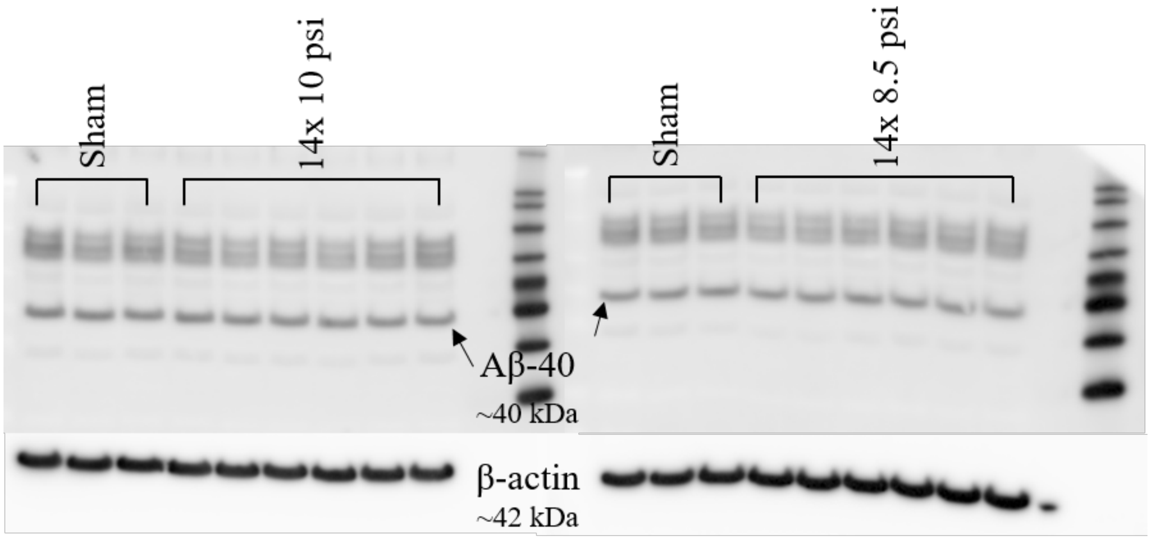

Supplementary Figure 6: One full Western blot of Aβ40 and β-actin.

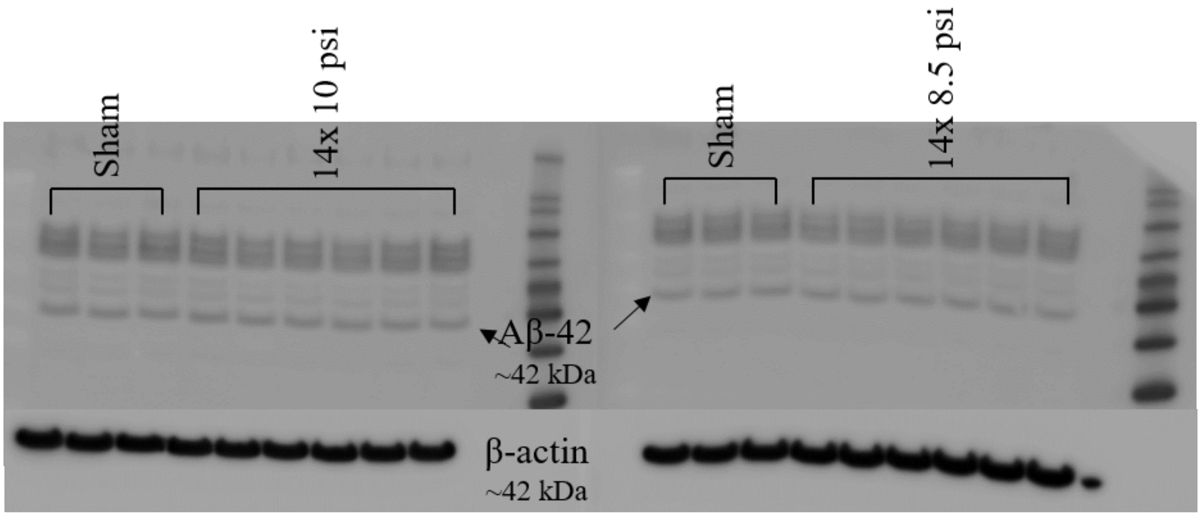

Supplementary Figure 7: One full Western blot of Aβ42 and β-actin.

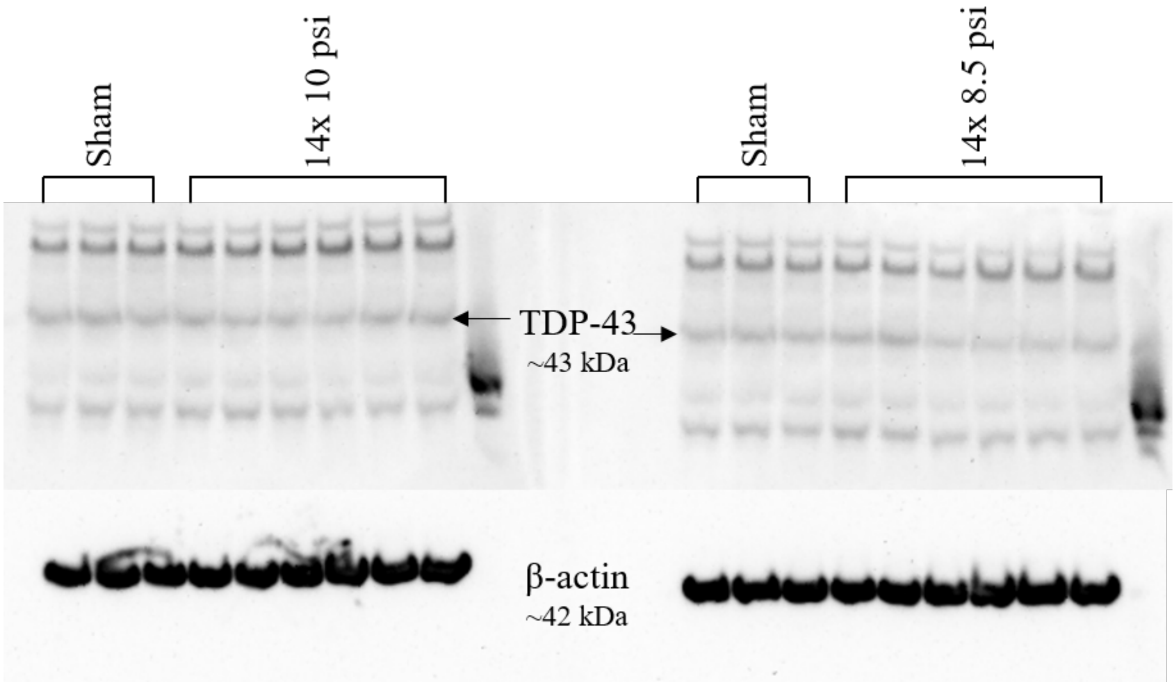

Supplementary Figure 8: One full Western blot of TDP-43 and β-actin.

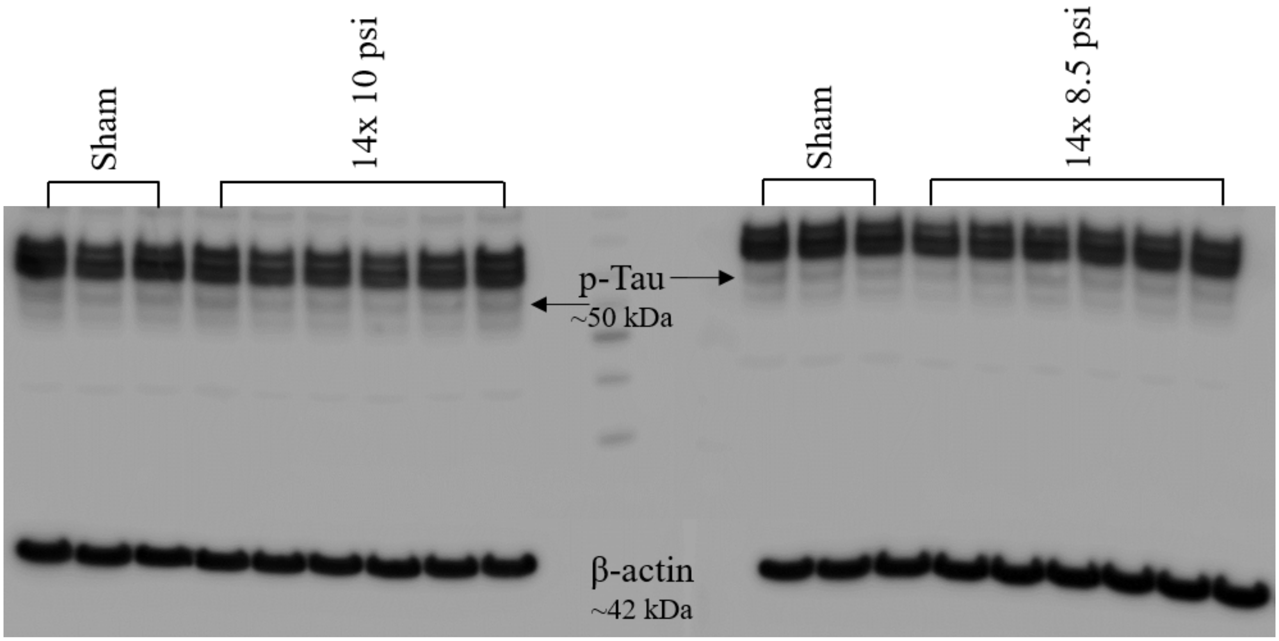

Supplementary Figure 9: One full Western blot of pTau and β-actin.
